# Immune responses and clinical outcomes following the third dose of SARS-CoV-2 mRNA-BNT162b2 vaccine in advanced breast cancer patients receiving targeted therapies: a prospective study

**DOI:** 10.3389/fonc.2023.1280416

**Published:** 2023-11-07

**Authors:** Fabrizio Nelli, Agnese Fabbri, Andrea Botticelli, Diana Giannarelli, Eleonora Marrucci, Cristina Fiore, Antonella Virtuoso, Julio Rodrigo Giron Berrios, Simone Scagnoli, Simona Pisegna, Alessio Cirillo, Valentina Panichi, Annalisa Massari, Maria Assunta Silvestri, Enzo Maria Ruggeri

**Affiliations:** ^1^ Department of Oncology and Hematology, Medical Oncology and Breast Unit, Central Hospital of Belcolle, Viterbo, Italy; ^2^ Department of Radiological, Oncological and Pathological Science, Sapienza University of Rome, Rome, Italy; ^3^ Biostatistics Unit, Scientific Directorate, Fondazione Policlinico Universitario A. Gemelli, Istituto di Ricovero e Cura a Carattere Scientifico (IRCCS), Rome, Italy; ^4^ Department of Oncology and Hematology, Cytofluorimetry Unit, Central Hospital of Belcolle, Viterbo, Italy; ^5^ Department of Oncology and Hematology, Pathology Unit, Central Hospital of Belcolle, Viterbo, Italy; ^6^ Department of Oncology and Hematology, Microbiology and Virology Unit, Central Hospital of Belcolle, Viterbo, Italy

**Keywords:** COVID-19 vaccine, third dose, SARS-CoV-2 breakthrough infections, immune response, breast cancer, CDK4/6 inhibitors, anti-HER2 therapies

## Abstract

**Purpose:**

Metastatic breast cancer patients are the most prevalent oncology population with advanced disease facing COVID-19 pandemic. Immune responses after mRNA-based vaccination during treatment with CDK4/6 inhibitors or HER2-directed agents remain unclear. We conducted a prospective analysis to elucidate changes in antibody titers and lymphocyte counts following full course of mRNA-BNT162b2 (tozinameran) vaccination in recipients undergoing these targeted therapies.

**Methods:**

Patients who had received a booster dosing and had been treated for at least 6 months were eligible. Antibody titers against SARS-CoV-2 spike protein were measured at four subsequent time points. Immunophenotyping of circulating lymphocytes was performed before the third dose of tozinameran and four weeks later to quantify the absolute counts of CD3^+^CD4^+^ T-helper cells, CD3^+^CD8^+^ T-cytotoxic cells, CD19^+^ B cells, and CD56^+^CD16^+^ NK cells. We also assessed the incidence of breakthrough infections and investigated whether immune changes affect time-to-treatment failure (TTF) after booster vaccination.

**Results:**

The current analysis included 69 patients, of whom 38 (55%) and 31 (45%) were being treated with CDK4/6 inhibitors and HER2-targeted therapies, respectively. All participants received a third dose of tozinameran between September 23 and October 7, 2021. Multivariate analysis revealed that CDK4/6 inhibition predicted a significantly impaired humoral response after the booster dose. This detrimental effect was also evident for T-helper cell counts before the third immunization, but it disappeared in the subsequent evaluation. After a median follow-up of 22.3 months, we observed 19 (26%) cases of COVID-19 outbreaks, all experiencing favorable clinical outcomes. Univariate analysis showed a significant association between the onset of SARS-CoV-2 infections and the use of CDK4/6 inhibitors, as well as with an impaired antibody and T-helper cell response. Only the last two covariates remained independent predictors after multivariate testing. Dynamic variations in antibody titers and T-helper cell counts did not affect TTF in multivariate regression analysis.

**Conclusions:**

Our results confirm that the immune response to tozinameran is impaired by CDK4/6 inhibitors, increasing the odds of breakthrough infections despite the third vaccine dose. Current evidence recommends maintaining efforts to provide booster immunizations to the most vulnerable cancer patients, including those with advanced breast cancer undergoing CDK4/6 inhibition.

## Introduction

The World Health Organization (WHO) has recently claimed that COVID-19 no longer constitutes a public health emergency of international concern. The same expert panel also recommended maintaining efforts to increase vaccine coverage against SARS-CoV-2 for all vulnerable individuals, including most cancer patients with advanced disease (1). Additional immunizations with mRNA-based vaccines were able to induce a more intense humoral and adaptive response than initial two-dose series (2). The enhanced immunity would prevent major sequelae from SARS-CoV-2 breakthrough infections, at least in the short term (3, 4). However, population-based studies have found that cancer patients continue to be at increased risk of COVID-19 outbreaks even after booster vaccinations, due to waning immune responses and the continuous surge of immune-evading variants of concern (5). The magnitude of breakthrough infection odds varies significantly among vaccinated patients, depending on specific cancer types and different active treatments (6). Since it is the most widespread tumor in women and the fifth cause of cancer mortality globally, breast cancer is likely the most prevalent malignancy that requires mitigation strategies against COVID-19 outbreaks (7). Vaccinated patients diagnosed with breast cancer were reported to have improved mortality rates and reduced severity of COVID-19 compared with their unvaccinated counterparts (8). Currently, breast cancer patients receive different kinds of therapy, with monoclonal antibodies against human epidermal growth factor receptor 2 (HER2) and cyclin-dependent kinase 4/6 (CDK4/6) inhibitors being the most commonly used targeted agents (9, 10). While limited data are available on HER-2-targeted therapies (11), the relationship between CKD4/6 inhibitors and the efficacy of vaccination remains controversial and concerns only the first or second immunization (12–16). Secondary neutropenia and lymphopenia occurring in most patients on CDK4/6 inhibition may hamper immune responses to vaccination (17). Conversely, preclinical and early clinical data suggest that exposure to CDK4/6 inhibitors significantly increases T cell activation, which could boost vaccine-induced immunity (18, 19). We performed a prospective subgroup analysis of the Vax-On-Third-Profile study to investigate longitudinal changes in antibody titers following full-course of mRNA-BNT162b2 (tozinameran) immunization in patients with advanced breast cancer receiving CDK4/6 inhibitors or HER2-targeted agents. Peripheral blood lymphocytes were also evaluated to determine whether their dynamic changes after the third dose of tozinameran affected clinical outcomes during exposure to the same treatments.

## Methods

### Study design and participants

We have already described the primary results of the Vax-On-Third-Profile study (clinical trial identifier: EudraCT number 2021-002611-54) (20). The study complied with the Strengthening the Reporting of Observational Studies in Epidemiology (STROBE) standards and was approved by the referring Ethics Committee (protocol number: 1407/CE Lazio1). All participants gave written informed consent before any procedure was performed. The current investigation was a predefined subgroup analysis that included patients with a histological diagnosis of metastatic breast cancer undergoing CDK4/6 inhibitors or HER2-targeted agents as either first- or later-line treatment. All patients were required to have received the third dose of tozinameran six months after the initial two-dose series. Eligible participants had to be on active treatment during the entire immunization schedule, from receipt of the first dose until at least the third dose of tozinameran. The absence of disease progression at restaging in the eight weeks prior to the third dose and at least one subsequent reassessment performed within six months were additional inclusion criteria. Evidence of previous SARS-CoV-2 infection at any time and receipt of cytotoxic chemotherapy in the four weeks preceding the third dose were exclusion criteria. Participants were tested for measuring IgG antibody levels against the SARS-CoV-2 spike protein (RBD-S1) and lymphocyte subpopulation counts. The development of SARS-CoV-2 breakthrough infections was monitored at different time points (3, 6, and 12 months) or whenever it occurred first following the completion of vaccination schedule. The primary endpoint was the assessment of humoral and lymphocyte responses in the cohort of patients on CDK4/6 inhibition (experimental cohort) in comparison with those receiving anti-HER2 direct therapies (control cohort). The secondary endpoints were to investigate the influence of immune responses on the occurrence of breakthrough infections and their effect on the failure of cancer treatment from any reason. In addition, the research evaluated survival outcome by time to treatment failure (TTF), which measures the length of time from the administration of the booster dose to the final discontinuation of active treatment for whatever cause. The interim analysis censored patients who had not withdrawn from cancer therapy (cut-off date June 30, 2023).

### Serologic and microbiologic assessments

Throughout the study, blood samples were taken at four subsequent points in time for serological testing (timepoint-1, three weeks after the initial dose; timepoint-2, four weeks following the second dose; timepoint-3, immediately prior to the third dose was administered; and timepoint-4, four weeks following the third dose). The titer of anti-RBD-S1 IgG antibodies was determined through the use of the SARS-CoV-2 IgG II Quant assay conducted on the ARCHITECT i2000sr automated platform provided by Abbott Laboratories, Diagnostics Division, Sligo, Ireland. The procedure was performed according to the manufacturer’s instructions as referenced (21). Initially, the results were expressed in arbitrary units per milliliter (AU/mL) over a linear range that was expanded to 80000 AU by an automated dilution. The serological titers obtained were then converted from AU to binding antibody units (BAU) after WHO International Standards for anti-SARS-CoV-2 immunoglobulin testing were released (1 Abbott AU corresponds to 0.142 WHO BAU) (22). Peripheral lymphocyte subsets were examined at time points 3 and 4 using the BD FACSCanto II system and BD FACSCanto clinical software (BD Biosciences, San Jose, CA), as outlined by the manufacturer (23). The panel used for staining included CD3 FITC, CD4 PE-Cy7, CD8 APC-Cy7, CD19 APC, CD45 PerCP-Cy5.5, CD56 PE, and CD16 PE (all from BD Biosciences). As we have already described, the BD Multitest 6-color TBNK reagent allowed us to quantify the absolute counts of T helper cells (CD3^+^CD4^+^), T cytotoxic cells (CD3^+^CD8^+^), B cells (CD19+), and NK cells (CD56^+^CD16^+^) (24). The results were presented as absolute cell counts/µL for each lymphocyte subset. Breakthrough infections were defined as laboratory-confirmed SARS-CoV-2 positivity by third-generation antigenic or polymerase chain reaction tests. Commercially available diagnostic assays were used according to standard public health protocols. All positive cases were reported to the government agency for epidemiological monitoring (25).

### Statistical analysis

Normally distributed variables were described using a mean with standard deviation, while skewed variables were described using a median with a 95% confidence interval or interquartile range (IQR). The Mann-Whitney *U* test for continuous variables and the Pearson’s *χ^2^* test for categorical data allowed for comparative evaluations. Comparisons between matched samples were carried out using the Wilcoxon signed-rank test or the McNemar test. We conducted a multivariate analysis of antibody titers and lymphocyte subset counts by fitting a linear generalized model on their logarithmic (log) values before and after booster dosing as a function of predefined covariates. Based on a receiver operating characteristic (ROC) curve calculated at the same time points, we evaluated the sensitivity and specificity of antibody titers and lymphocyte subset counts in predicting the likelihood of breakthrough infections. For subsequent analyses, we deemed immune parameters relevant if they showed a statistically significant association with the intended outcome. The Youden index was applied to determine the optimal cut-point. A multivariate logistic regression model was implemented to estimate the odds ratio (OR) of breakthrough infections with a 95% CI in relation to the significant variables. A Mantel-Cox log-rank test allowed for comparison of survival outcomes between predefined patient subgroups. The Kaplan-Meier method was used to visualize survival curves. To calculate the hazard ratio (HR) with a 95% CI of confirmed significant variables, a multivariate Cox regression model was applied. The tests were all two-sided, and a significant *P* value was defined as less than 0.05. All statistical evaluations and figure rendering were performed using SPSS (IBM SPSS Statistics for Windows, version 23.0, Armonk, NY) and Prism (GraphPad, version 9), respectively.

## Results

### Patient characteristics

Out of the 258 patients initially enrolled, 69 were eligible for the current analysis, of whom 38 (55%) and 31 (45%) were being given with CDK4/6 inhibitors and anti-HER2 therapies, respectively. All participants were administered a third dose of tozinameran from September 23 to October 7, 2021. The median age was 64 years and all recipients had a metastatic disease with an Eastern Cooperative Oncology Group Performance Status (ECOG PS) of 0 to 1. Most clinical variables were evenly distributed across the treatment cohorts. A negative hormone receptor status and receipt of previous cytotoxic chemotherapy were significantly more frequent in patients receiving anti-HER2 therapies owing to the intrinsic molecular features of their breast cancer subtype. The median length of treatment overall and before the third dose of tozinameran did not differ significantly between the cohorts. **Table 1** depicts in detail the baseline characteristics of the enrolled patients.

**Table 1:** Patient characteristics.

Characteristic	General population, N=69 (100%)	CKD4/6 inhibitor cohort, N=38 (100%)	Anti-HER2 therapy cohort, N=31 (100%)	P value
Mean age, years (SD) - ≥65 years	63.7 (10.9) 37 (46.4%)	64.3 (10.29 21 (55.2%)	63.0 (11.7) 16 (51.6%)	0.681 0.952
Sex - female	69 (100.0%)	38 (100.0%)	31 (100.0%)	
Menopausal status - premenopausal - postmenopausal	16 (23.2%) 53 (76.8%)	8 (21.0%) 30 (79.0%)	8 (25.8%) 23 (74.2%)	0.858
Smoking habits - never - ever	41 (59.4%) 28/40.6%)	22 (57.9%) 16 (42.1%)	19 (61.3%) 12 (37.8%)	0.969
BMI - mean (SD) - >25 kg/m^2^	24.9 (4.3) 34 (49.3%)	24.9 (3.6) 19 (50.0%)	24.9 (5.0) 15 (48.4%)	0.754 0.894
Histology - lobular - ductal	10 (14.5%) 59 (85.5%)	5 (13.1%) 33 (86.9%)	5 (16.1%) 26 (83.9%)	0.996
Hormone receptor status - negative - positive	6 (8.7%) 63 (91.3%)	38 (100.0%)	6 (19.3%) 25 (80.7%)	0.016
KI67 scoring - <20% - ≥20%	17 (24.6%) 52 (75.4%)	8 (21.0%) 30 (79.0%)	9 (29.0%) 22 (71.0%)	0.628
Number of metastatic sites - 1 - ≥2	37 (53.6%) 32 (46.4%)	16 (41.1%) 22 (57.9%)	21 (67.7%) 10 (32.3%)	0.060
Visceral involvement - not present - any	46 (66.7%) 23 (33.3%)	27 (71.0%) 11 (29.0%)	19 (61.3%) 12 (38.7%)	0.549
ECOG PS - 0 - 1	45 (65.2%) 24 (34.8%)	21 (55.3%) 17 (44.7%)	24 (77.4%) 7 (22.6%)	0.095
Treatment setting - metastatic, first line - metastatic, second or later line	47 (68.1%) 22 (31.9%)	24 (63.2%) 14 (36.8%)	23 (74.2%) 8 (25.8%)	0.472
CDK4/6 inhibitor - palbociclib - ribociclib - abemaciclib	18 (26.1%) 15 (21.7%) 5 (7.2%)	18 (47.4%) 15 (39.5%) 5 (13.1%)		
Anti-HER2 therapy - trastuzumab only - trastuzumab and pertuzumab - T-DM1	17 (24.6%) 7 (10.1% 7 (10.1%)		17 (54.8%) 7 (22.6%) 7 (22.6%)	
Previous cytotoxic chemotherapy - none - any	8 (11.6%) 61 (88.4%)	38 (100.0%)	23 (74.2%) 8 (25.8%)	0.003
Overall length (months) of treatment, median (IQR)	32.9 (26.4-48.4)	32.3 (22.7-45.4)	34.6 (28.1-56.4)	0.102
Length (months) of treatment before third vaccine dose, median (IQR)	14.5 (9.8-34.4)	15.6 (10.6-31.8)	13.8 (8.7-44.4)	0.819

CDK, cyclin-dependent kinase; HER2, epidermal growth factor receptor 2; SD, standard deviation; BMI, body mass index; ECOG PS, Eastern Cooperative Oncology Group Performance Status; T-DM1, trastuzumab emtansine, IQR, interquartile range.

Previous cytotoxic chemotherapy indicate receipt in the time frame between the first and the third dose tozinameran.

### Antibody responses

**Figure 1:**
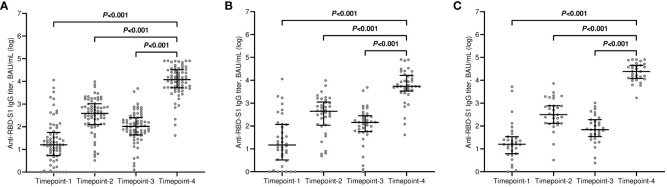
Longitudinal variations in antibody titers. **(A)** general population; **(B)** CDK4/6 inhibitor cohort; **(C)** anti-HER2 therapy cohort. RBD-S1, receptor-binding domain (RBD) of the SARS-CoV-2 Spike protein (S1); BAU, Binding Antibody Unit; log, logarithmic values. Bars represent median values with interquartile range. Timepoint-1 denotes assessment three weeks after the first dose of tozinameran; timepoint-2 denotes assessment eight weeks after the second dose of tozinameran; timepoint-3 denotes assessment before the third dose of tozinameran; timepoint-4 denotes assessment four weeks after the third dose of tozinameran.

**Figure 2:**
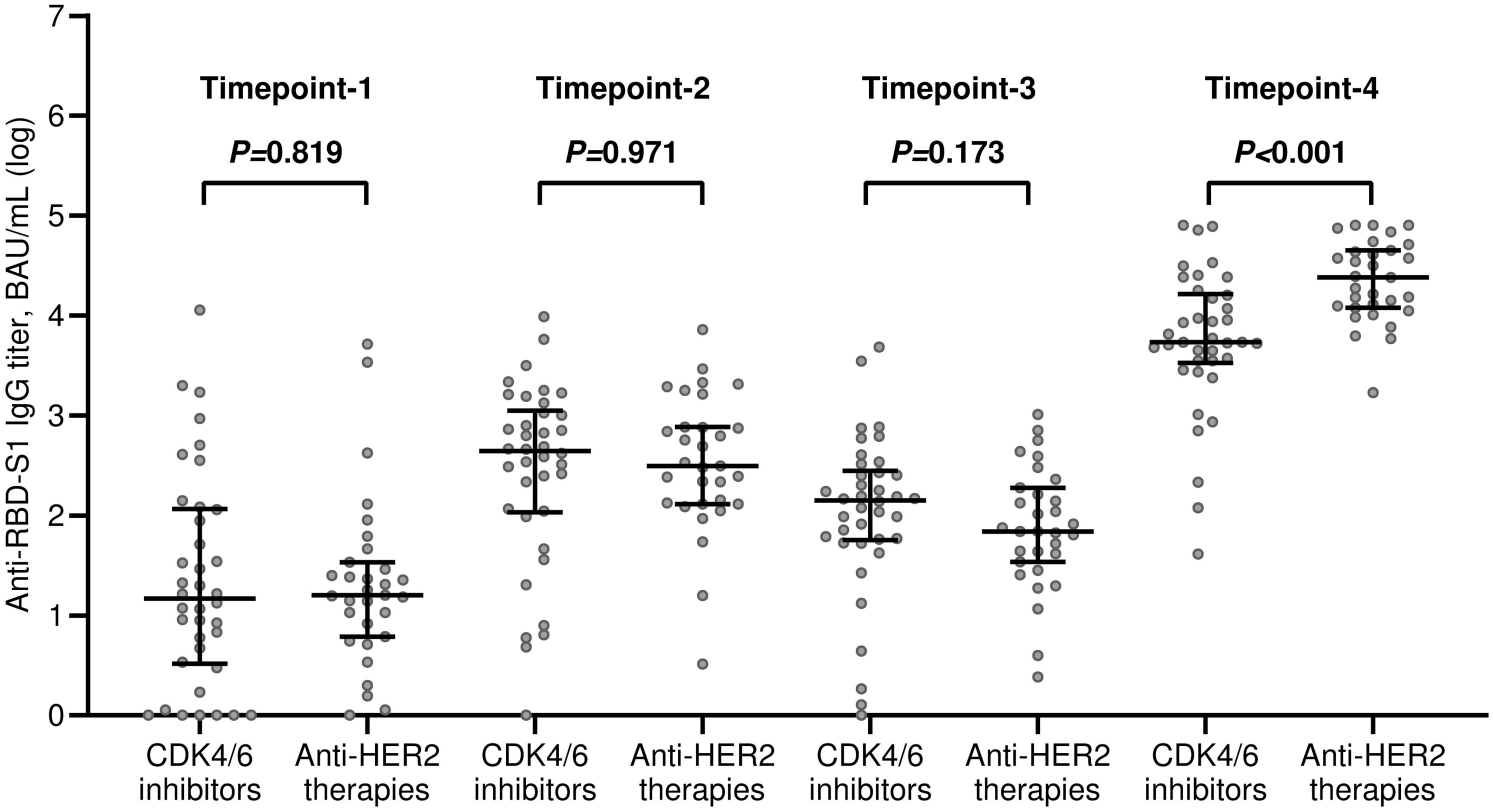
Univariate comparison of changes in antibody titers. RBD-S1, receptor-binding domain (RBD) of the SARS-CoV-2 Spike protein (S1); BAU, Binding Antibody Unit; log, logarithmic values. Bars represent median values with interquartile range. Timepoint-1 denotes assessment three weeks after the first dose of tozinameran; timepoint-2 denotes assessment eight weeks after the second dose of tozinameran; timepoint-3 denotes assessment before the third dose of tozinameran; timepoint-4 denotes assessment four weeks after the third dose of tozinameran.

The median value of the antibody titers increased exponentially after the third dose of tozinameran and was significantly higher than the appraisals obtained at previous time points (**Figure 1A**). This incremental change occurred in both treatment cohorts (**Figures 1B, C**). While the univariate comparison between treatment cohorts showed no difference in antibody responses across the longitudinal evaluation before the booster dose, patients receiving CDK4/6 inhibitors experienced a significant decrease in their titers at timepoint-4 (**Supplementary Table 1**, **Figure 2**). Multivariate analysis according to predefined clinical covariates revealed that only treatment line (second or subsequent line vs. first line) had a significant effect on antibody levels before the third dose of tozinameran (**Supplementary Table 1**). The same multivariate analysis model showed that the type of targeted treatment (CDK4/6 inhibitors vs. anti-HER2 therapies) was the most reliable predictor of antibody responses after the third immunization (**Supplementary Table 2**).

### Changes in circulating lymphocytes

**Figure 3:**
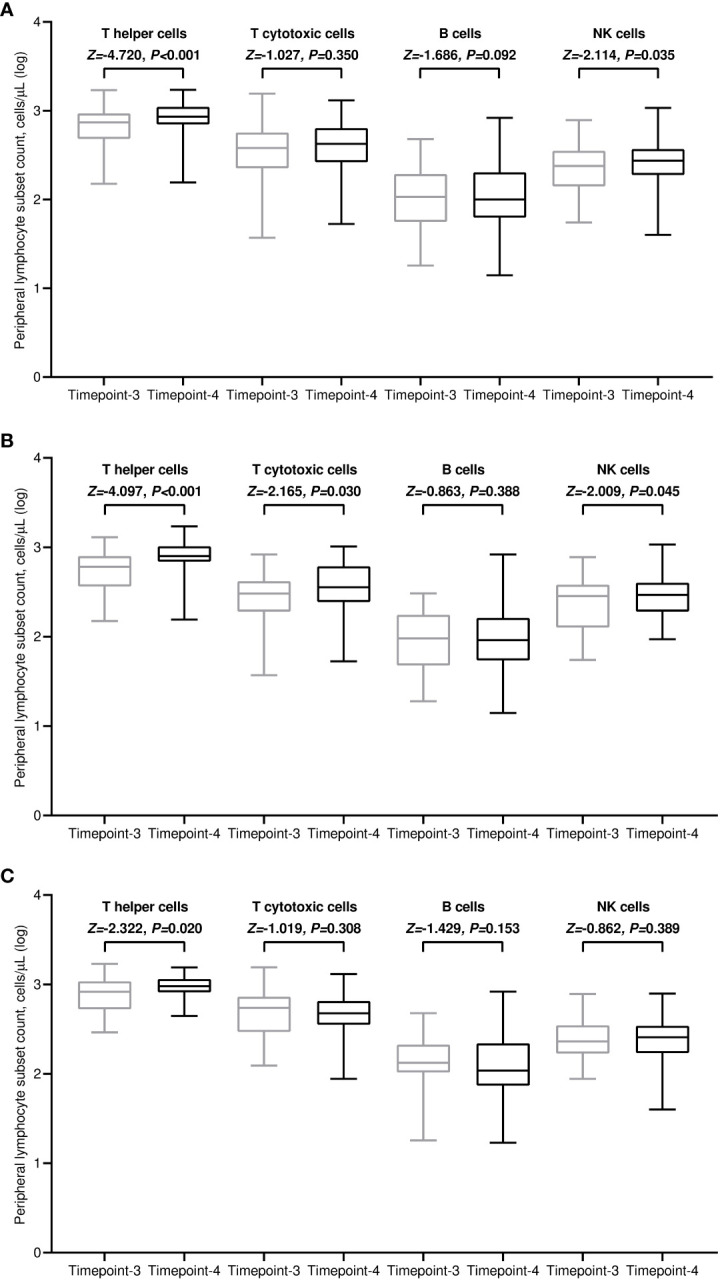
Dynamic variations in peripheral lymphocyte subpopulations. **(A)** general population; **(B)** CDK4/6 inhibitor cohort; **(C)** anti-HER2 therapy cohort. Log, logarithmic value; T helper cells, CD3^+^CD4^+^ cells; T cytotoxic cell, CD3^+^CD8^+^; B cells, CD19^+^; NK, Natural killer, CD56^+^CD16^+^; timepoint-3 denotes assessment before the third dose of tozinameran; timepoint-4 denotes assessment four weeks after the third dose of tozinameran.

**Figure 4:**
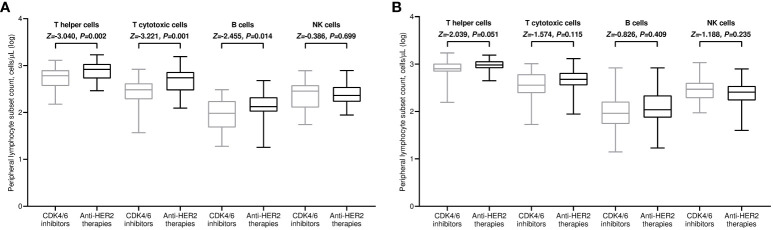
Univariate comparison of changes in peripheral lymphocyte subpopulations. **(A)** Comparison at timepoint-3; **(B)** comparison at timepoint-4. Log, logarithmic value; T helper cells, CD3^+^CD4^+^ cells; T cytotoxic cell, CD3^+^CD8^+^; B cells, CD19^+^; NK, Natural killer, CD56^+^CD16^+^; timepoint-3 denotes assessment before the third dose of tozinameran; timepoint-4 denotes assessment four weeks after the third dose of tozinameran.

All included patients completed a flow cytometry assessment of peripheral blood at both time points. Within the general population, the median values of all lymphocyte subpopulations increased after the third immunization in comparison with baseline. However, this incremental variation was statistically significant only for T helper and NK cell subsets (**Figure 3A**). The rise in T helper cell absolute counts was valuable in both treatment cohorts (**Figures 3B, C**). It is noteworthy to observe that patients receiving CDK4/6 inhibitors had significantly decreased levels of T helper, T cytotoxic, and B cells before the third vaccination compared to those given with anti-HER2 therapies (**Figure 4A**). The significant value of this difference was not confirmed in the same comparison following the booster dose of tozinameran (**Figure 4B**). Multivariate analysis revealed that the type of targeted therapy had a strong independent effect on T and B cell counts tested before the booster dose (**Supplementary Table 3**). This covariate had no impact on any subpopulation of immune cells after the third immunization (**Supplementary Table 4**).

### Breakthrough infections

**Figure 5:**
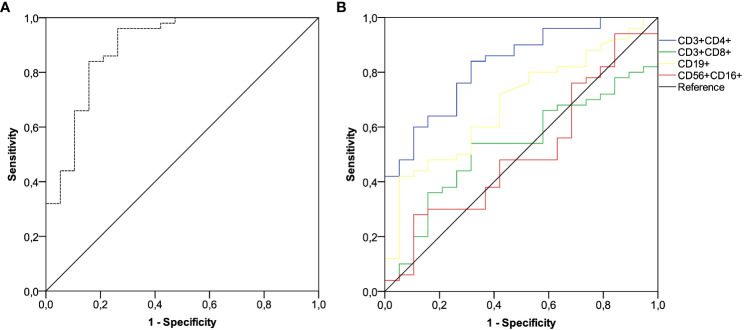
ROC curve analysis. **(A)** ROC curve analysis showing the performance of absolute anti-RBD-S1 IgG titers in predicting protection from SARS-CoV-2 breakthrough infection at timepoint-4; AUC relative value: 0.894 (95% CI 0.800-0.988), *P*<0.001. **(B)** ROC curve analysis showing the performance of absolute counts of peripheral lymphocyte subsets in predicting protection from SARS-CoV-2 breakthrough infection at timepoint-4; AUC relative values: T helper cells (CD3^+^CD4^+^): 0.828 (95% CI 0.727-0.930; *P*<0.001); T cytotoxic cells (CD3^+^CD8^+^): 0.529 (95% CI 0.388-0.671; *P*=0.707); B cells (CD19^+^): 0.686 (95% CI 0.553-0.818; *P*=0.018); NK cells (CD56^+^CD16^+^): 0.506 (95% CI 0.354-0.658; *P*=0.936). Timepoint-4 indicates assessment four weeks after the third dose of tozinameran. ROC, receiver operating characteristic; RBD-S1, SARS-CoV-2 receptor binding domain spike protein; AUC, area under the curve; CI, confidence interval.

After a median follow-up time of 22.3 months (95% CI 18.1-23.7), 19 of 69 (25.7%) patients developed a SARS-CoV-2 breakthrough infection with a median interval of 2.9 months (IQR 1.8-4.1) following the third immunization. Most patients were asymptomatic or mildly symptomatic. Three cases (15.8%) showed moderate symptoms that required home antiviral therapy. Only one patient (5.2%) exhibited a severe clinical course that prompted hospital admission. No deaths related to COVID-19 outbreaks were observed. A primary ROC curve was calculated to determine the relationship between anti-RBD-S1 IgG titers after booster dosing and protection from SARS-CoV-2 outbreaks. The relative AUC value [0.89 (95% CI 0.80-0.98), *P*<0.001] was considered valuable in predicting the likelihood of a negative outcome (**Figure 5A**). The Youden index identified an optimal IgG titer cut-point of 907 BAU/mL, which was associated with a sensitivity of 0.84 and a specificity of 0.85 and allowed dichotomization of recipients into low antibody-responders (<907 BAU/mL) and high antibody-responders (≥907 BAU/mL). We computed a secondary ROC curve to determine the relationship between absolute counts of peripheral lymphocyte subpopulations after booster dosing and the avoidance of SARS-CoV-2 breakthrough infections. The relative value of AUC pertaining to T helper cell distribution [0.82 (95% CI 0.72-0.93), *P*<0.001] was considered meaningfully related to the probability of a negative outcome (**Figure 5B**). In this case, the Youden index indicated a count of 806/µL as the optimal cut-point for T helper cell distribution. This threshold value yielded a sensitivity of 0.76 and a specificity of 0.74, allowing the recipients to be divided into distinct subgroups of low T helper-responders (<806/µL) and high T helper responders (≥806/µL). Univariate analysis demonstrated a significant association between the onset of SARS-CoV-2 infection and the use of CDK4/6 inhibitors, as well as an impaired antibody and T helper cell response. Only the last two covariates remained independent predictors after multivariate testing (**Table 2**).

**Table 2:** Analysis of breakthrough infections.

Covariate	Univariate analysis		Multivariate analysis	
	Breakthrough infections	P value	OR (95% CI)	P value
Age - <65 years (N=32) - ≥65 years (N=37)	9 (28.1%) 10 (27.0%)	0.918		
Menopausal status - premenopausal (N=16) - postmenopausal (N=53)	4 (25.0%) 15 (28.3%)	0.796		
Smoking habits - never (N=41) - ever (N=28)	11 (26.8%) 8 (28.6%)	0.874		
BMI (kg/m^2^) - ≤25 (N=35) - >25 (N=34)	8 (22.9%) 11 (32.4%)	0.377		
Histology - lobular (N=10) - ductal (N=59)	3 (30.0%) 16 (27.1%)	0.850		
Hormone receptor status - negative (N=6) - positive (N=63)	19 (30.2%)	0.114		
KI67 scoring - <20% (N=17) - ≥20% (N-=52)	6 (35.3%) 13 (25.0%)	0.406		
Number of metastatic sites - 1 (N=37) - ≥2 (N=32)	12 (32.4%) 7 (21.69%)	0.478		
Visceral involvement - not present (N=46) - any (N=23)	13 (28.3%) 6 (26.1%)	0.849		
ECOG PS - 0 (N=45) - 1 (N=24)	13 (28.9%) 6 (25.0%)	0.731		
Treatment setting - metastatic, first line (N=47) - metastatic, second or later line (N=22)	13 (27.7%) 6 (27.3%)	0.973		
Previous cytotoxic chemotherapy^a^ - none (N=61) - any (N=8)	18 (29.5%) 1 (12.5%)	0.311		
Targeted treatment - anti-HER2 therapies (N=31) - CDK4/6 inhibitors (N=38)	15 (39.5%) 4 (12.9%)	0.014	1.00 1.18 (0.46-3.04)	0.719
Antibody response^b^ - high responders (N=45) - low responders (N=24)	3 (6.7%) 16 (66.7%)	<0.001	1.00 4.68 (1.94-11.28)	0.001
T helper cell response^c^ - high responders (N=43) - low responders (N=26)	4 (11.6%) 15 (53.8%)	<0.001	1.00 2.16 (1.01-4.43)	0.045

OR, odds ratio; CI, confidence interval; BMI, body mass index; ECOG PS, Eastern Cooperative Oncology Group Performance Status; CDK, cyclin-dependent kinase; HER2, epidermal growth factor receptor 2.

a Previous cytotoxic chemotherapy indicate receipt in the time frame between the first and the third dose tozinameran.

b low-responders indicate the subgroup of patients with an anti-RBD-S1 IgG titer <907 BAU/mL after the third dose of vaccine, high-responders indicates the subgroup of patients with an anti-RBD-S1 IgG titer ≥907 BAU/mL after the third dose of vaccine.

c low-responders indicate the subgroup of patients with T helper cell count <806/µL after the third dose of tozinameran, high-responders indicate the subgroup of patients with NK cell count ≥806/µL after the third dose of tozinameran.

### Survival outcomes

**Figure 6:**
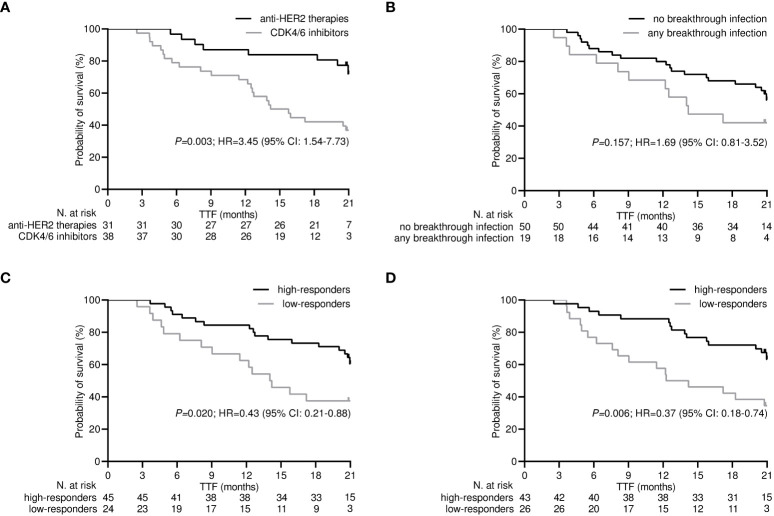
Time-treatment-failure after the third dose of tozinameran. **(A)** type of targeted therapy: anti-HER2-therapies vs. CDK4/6 inhibitors; **(B)** breakthrough infection: not vs. yes; **(C)** level of antibody response: high-responders (subgroup of patients with an anti-RBD-S1 IgG titer ≥907 BAU/mL after the third dose of vaccine) vs low-responders (subgroup of patients with an anti-RBD-S1 IgG titer <907 BAU/mL after the third dose of vaccine); **(D)** level of T helper cell level: high-responders (subgroup of patients with T helper cell count ≥806/µL after the third dose of vaccine) vs. low-responders (subgroup of patients with T helper cell count <806/µL after the third dose of vaccine).

After a median follow-up time of 21.0 months (95% CI 19.8-22.1), 32 (46.4%) patients withdrew from targeted treatments and 37 (53.6%) were censored during active therapy owing to the lack of TTF-relevant events as of the last interim analysis. The rate of patients who discontinued treatment for any reason was significantly higher for those receiving CDK4/6 inhibitors compared with HER2-targeted therapies (63.2% vs. 25.8%, *P*=0.002). Univariate survival analysis showed that treatment with CDK4/6 inhibitors and a low level of antibody and T helper response after the third dose of tozinameran were associated with an increased risk of treatment failure (**Table 3**, **Figure 6**). However, only the type of targeted therapy retained its significant potential for TTF in the multivariate analysis (**Table 3**).

**Table 3:** Analysis of time-to-treatment failure after the third dose of tozinameran.

Covariate	Time-to-treatment failure				
	Univariate analysis			Multivariate analysis	
	Median TTF (95% CI), months	HR (95% CI)	P value	HR (95% CI)	P value
Treatment type - anti HER2 therapies - CDK4/6 inhibitors	20.8 (17.2-NR) 14.2 (11.0-17.3)	1.00 3.45 (1.54-7.73)	0.003	1.00 2.57 (1.06-6.22)	0.035
Breakthrough infection - none - yes	20.5 (12.7-28.3) 12.5 (6.1-18.8)	1.00 1.69 (0.81-3.52)	0.157	1.00 0.83 (0.34-2.05)	0.696
Antibody level^a^ - high-responders - low-responders	20.7 (16.2-NR) 14.7 (9.7-18.3)	1.00 0.43 (0.21-0.88)	0.020	1.00 0.76 (0.33-1.78)	0.541
T helper level^b^ - high-responders - low-responders	18.1 (16.5-NR) 12.2 (5.0-19.4)	1.00 0.37 (0.18-0.74)	0.006	1.00 0.53 (0.22-1.27)	0.160

CI, confidence interval; HR, hazard ratio; CDK, cyclin-dependent kinase; HER2, epidermal growth factor receptor 2; NR, not reached.

a low-responders indicate the subgroup of patients with an anti-RBD-S1 IgG titer <907 BAU/mL after the third dose of vaccine, high-responders indicates the subgroup of patients with an anti-RBD-S1 IgG titer ≥907 BAU/mL after the third dose of vaccine;

b low-responders indicate the subgroup of patients with T helper cell count <806/µL after the third dose of tozinameran, high-responders indicate the subgroup of patients with NK cell count ≥806/µL after the third dose of tozinameran

## Discussion

This predefined subgroup analysis of the Vax-On-Third-Profile primarily investigated the immune responses of patients with advanced breast cancer who received booster doses of tozinameran while undergoing targeted treatments. We focused on recipients who were receiving CDK4/6 inhibitors, as their immune reactogenicity was expected to be impaired compared to those given with anti-HER2-directed therapies. Longitudinal assessment of antibody titers revealed a blunted humoral response for CDK4/6 inhibition throughout the entire study period. In these patients, the third dose of vaccine resulted in a favorable response for T helper cell counts, flattening the significant difference observed before booster immunization. We secondarily evaluated the clinical implications of the changes in systemic immunity induced by the third vaccination. Patients receiving CDK4/6 inhibitors were more likely to contract SARS-CoV-2 breakthrough infections, which, however, did not result in any serious adverse events. Dynamic variations in humoral and lymphocyte responses relevant to COVID-19 outbreaks did not affect the likelihood of treatment failure after booster dosing. Similar immunologic findings have not been reported previously and raise several matters for discussion.

A first key issue concerns the choice of targeted therapies for advanced breast cancer with regard to their relevance to the immune effects of COVID-19 vaccination. We leaned toward CKD4/6 inhibitors and HER2-targeted agents because they are the most frequently prescribed in this setting and their immunologic implications are well characterized. For the purpose of comparison in our experimental design, monoclonal antibodies directed against HER2 represented the control cohort. The interplay between the immune system and HER2-targeted treatments has recently moved into the spotlight, emerging as dynamic and complex (26). This interaction has been shown to be favorable in terms of immune surveillance and immune balance, making a detrimental effect on the response to anti-SARS-CoV-2 vaccination unlikely (27). Although a single study investigating inactivated BBIBP-CorV vaccine raised concerns about immunologic interference with trastuzumab (11), larger evidence on mRNA-based immunizations did not confirm this potential threat (28). Exposure to CDK4/6 inhibitors during the full vaccine schedule accounted for the experimental cohort because of several reasons. While neutropenia and lymphopenia are common adverse events with CDK4/6 inhibition, such therapy is not generally considered to be immunosuppressive. In fact, because myelotoxicity is induced by reversible suppression of bone marrow precursors through cell cycle arrest, the incidence of febrile neutropenia and related infections is very low (29). Downregulation of the CDK4/6 complex reduces the expression of specific DNA methyltransferase genes, which leads to hypomethylation and increased transcription of endogenous retroviral elements (30). These changes would have a positive impact on vaccination outcomes because they trigger a “viral mimicry” response, characterized by interferon production and expression of interferon-stimulated genes (31, 32). Nonetheless, several clinical trials have observed impaired humoral responses after an initial two-dose series for patients receiving CDK4/6 inhibitors (12–14). Regardless of the diversity of the control group in these studies (patients on endocrine therapy or healthy volunteers), the serological findings of the current research are widely consistent with the available evidence. In addition, we provided for the first time a comprehensive assessment of antibody titer changes after the booster dose of COVID-19 vaccination. Our univariate data confirm that the humoral response on CDK4/6 inhibition remains underpowered, which is supported by multivariate analyses showing an independent effect of targeted treatments.

Changes in lymphocyte responses after vaccine booster represent a second key issue in this research. Previous clinical studies have described a reduction in SARS-CoV-2-specific T helper and T cytotoxic cell reactivity after the second dose of mRNA-1273 vaccine in patients receiving CDK4/6 inhibitors (15, 33). Unlike these investigations, we performed a basic immunophenotypic characterization of peripheral blood, which provides a generic representation of lymphocyte dynamics after the third dose of tozinameran. Several studies have found a viable correlation between the outcomes of SARS-CoV-2-specific T and B cell assays and their absolute counts, supporting the validity of this approach for monitoring adaptive immunity in the context of COVID-19 vaccination (34, 35). While giving due consideration to methodological differences, our results are consistent with the described evidence, confirming that CDK4/6 inhibition hampers the T cell response six months after the initiation of mRNA-based vaccination. However, we observed that booster immunization was able to mitigate disparities in T cell-mediated responses, suggesting that this additional measure may overcome the detrimental effect of treatment with CDK4/6 inhibitors. The lack of an independent effect of targeted therapy on T lymphocyte levels after the third dose of tozinameran, evident instead in the multivariate assessment at baseline, would lend support to this hypothesis. Since, to the best of our knowledge, similar results have not been previously reported, no further insights can be drawn.

A third key question concerns the clinical implications of our immunological results regarding the odds of SARS-CoV-2 breakthrough infection and survival outcomes. Patients receiving CDK4/6 inhibitors had a significantly higher incidence of COVID-19 outbreaks during a prolonged period of longitudinal observation after booster vaccine dosing. Consistent with previous research, level of antibody and T helper cell response, but not specific targeted treatment, were independent risk factors for contracting breakthrough infections (15). It is noteworthy to observe that all infections had a favorable clinical course. This suggests that, despite the diversity in humoral and adaptive reactogenicity, the immunologic changes induced by additional immunization have a protective effect against severe COVID-19. We further investigated whether these immune responses influence disease outcomes in terms of time-to-treatment failure after the third dose of tozinameran. Treatment with CDK4/6 inhibitors exerts direct effects on T lymphocyte function in the tumor microenvironment (TME), including a decrease in the proliferative capacity of regulatory T cells and an increase in the activity of effector T cells (36, 37). These immune shifts result in the transition of TME to an inflammatory phenotype, which contributes to the antineoplastic efficacy of CDK4/6 inhibition regardless of cell cycle arrest (38). Conversely, recent evidence indicates that mRNA-based immunization may trigger biological processes disrupting the maintenance of immune competence and cellular homeostasis (39). In addition, vaccinations based on recombinant technology induce downregulation of IFNα signaling. Downstream events of this altered cytokine regulation could hinder cancer immunoediting and interfere with the immunologic effects of both CDK4/6 inhibitors and HER2-targeted therapies (40). Since protection from infections is the main purpose of any vaccination, we evaluated the level of antibody and lymphocyte responses as a function of this clinical end-point. Accordingly, dynamic changes in humoral and adaptive immunity did not affect the clinical efficacy of either targeted treatment on multivariate testing. The latter finding would imply that there was no interaction between the third dose of tozinameran and the immune effects of CDK4/6 inhibitors or monoclonal antibodies directed against HER2. However, the lack of comparable studies, as well as the unavailability of comparison with a control group of unvaccinated patients, does not allow further insights to be drawn.

The current study recognizes several constraints that include, but are not limited to, the following. The original study had only receipt of active cancer treatment and eligibility for the third dose of tozinameran as inclusion criteria. Although the present subgroup analysis relies on pre-planned research hypothesis, the all-comer recruitment involves inherent selection bias. The definition of the treatment cohorts was based on relevant clinical and immunological assumptions, but their choice remains arbitrary. As ideal as a control group of unvaccinated patients would have been, the unavoidability of COVID-19 vaccination precluded us from this comparison. The reliability of absolute counts of peripheral lymphocyte subsets as a correlate of adaptive immunity induced by mRNA vaccination is still controversial. We are aware that enzyme-linked immunosorbent spot (ELISpot) tests would have provided a more specific assessment of cell-mediated responses against SARS-CoV-2 (41). However, the low level of standardization and methodological challenges hinder clinical deployment of these assays (42, 43). Our multivariate analysis did not include the receipt of additional boosters beyond the third dose among the predictors of breakthrough infections. Because such preventive measures were authorized as of March 2022, when 37% of enrolled patients had already contracted COVID-19 outbreaks, this introduces confounding that was unpredictable at baseline (44). The assessment of survival outcome suffers from an implicit immortal time bias, which stems from the interval between the start of targeted therapies and the third dose of tozinameran (45). To mitigate the effects of this potential flaw, we referred to the TTF following the booster dose because it pertains to a homogeneous event over time for all patients. Finally, despite it overlaps with that of similar studies, the sample size of our research is otherwise small. This observation implies an increased risk of false-positive findings arising from multivariate comparisons, the significance of which should be considered suggestive of further research hypotheses.

## Conclusions

The epidemiological and clinical features of breast cancer diagnosis make these patients the most relevant oncology population facing the current and future implications of the COVID-19 pandemic. Despite evidence of a favorable safety profile (46), hesitation toward booster vaccinations remains higher than in the general population (47). Extensive studies have conclusively established that additional vaccinations provide the best protection against mortality and serious complications related to COVID-19 in high-risk recipients (48, 49). Our findings confirm that treatment with CDK4/6 inhibitors for advanced breast cancer impairs the immune response to tozinameran and is a risk factor for the development of breakthrough infections despite the third vaccine dose. The favorable course of all observed COVID-19 outbreaks and early survival data seem reassuring about the clinical efficacy of booster dosing. While shortcomings of this study warrant further validation, current evidence recommends maintaining efforts to provide booster immunizations to the most vulnerable breast cancer patients, including those undergoing CDK4/6 inhibition.

## Data availability statement

The raw data supporting the conclusions of this article will be made available by the authors, without undue reservation.

## Ethics statement

The studies involving humans were approved by Comitato Etico Lazio 1, Circonvallazione Gianicolense, Roma, Italy. The studies were conducted in accordance with the local legislation and institutional requirements. The participants provided their written informed consent to participate in this study.

## Author contributions

FN: Conceptualization, Data curation, Investigation, Methodology, Supervision, Validation, Visualization, Writing – original draft, Writing – review & editing. AF: Data curation, Investigation, Writing – original draft, Writing – review & editing. AB: Conceptualization, Validation, Writing – original draft, Writing – review & editing. DG: Formal Analysis, Methodology, Software, Writing – review & editing. EM: Data curation, Investigation, Writing – review & editing. CF: Data curation, Investigation, Writing – review & editing. AV: Data curation, Investigation, Writing – review & editing. JG: Data curation, Investigation, Writing – review & editing. SS: Data curation, Investigation, Writing – review & editing. SP: Data curation, Investigation, Writing – review & editing. AC: Data curation, Investigation, Writing – review & editing. VP: Investigation, Validation, Writing – review & editing. AM: Investigation, Validation, Writing – review & editing. MS: Investigation, Validation, Writing – review & editing. ER: Conceptualization, Methodology, Project administration, Supervision, Validation, Writing – original draft, Writing – review & editing.
