# Supplementary material for: Immune responses and clinical outcomes following the third dose of SARS-CoV-2 mRNA-BNT162b2 vaccine in advanced breast cancer patients receiving targeted therapies: a prospective study
Source: Front Oncol. 2023 Nov 7;13:1280416. doi: 10.3389/fonc.2023.1280416 (PMC10662103; doi:10.3389/fonc.2023.1280416)
Supplement: Supplementary file 1 [file DataSheet_1.docx]

**SUPPLEMENTARY MATERIAL**

Supplementary Table 1

Supplementary Table 2

Supplementary Table 3

Supplementary Table 4

Supplementary Figure 1

Supplementary Table 1. Univariate comparison of antibody titers

| Anti-RBD-S1 antibody titers (BAU/mL), median with 95% CI | General population | CDK4/6 inhibitor cohort | Anti-HER2 therapy cohort | P value |
| --- | --- | --- | --- | --- |
| Timepoint-1 | 15.9 (10.6-23.1) | 14.8 (7.9-33.5) | 15.9 (10.6-24.1) | 0.819 |
| Timepoint-2 | 388.5 (294.4-624.3) | 439.2 (262.1-710.8) | 313.6 (216.2-690.5) | 0.971 |
| Timepoint-3 | 103.1 (69.3-146.1) | 141.9 (82.3-177.9) | 69.4 (44.1-139.5) | 0.173 |
| Timepoint-4 | 1695.6 (1205.1-2323.5) | 769.1 (638.5-1313.2) | 3409.2 (1847.5-5353.4) | <0.001 |

RBD-S1, SARS-CoV-2 receptor binding domain spike protein; BAU, binding antibody unit; CDK, cyclin-dependent kinase; HER2, epidermal growth factor receptor 2; CI, confidence interval. Timepoint-1 denotes assessment three weeks after the first dose of tozinameran; timepoint-2 denotes assessment eight weeks after the second dose of tozinameran; timepoint-3 denotes assessment before the third dose of tozinameran; timepoint-4 denotes assessment four weeks after the third dose of tozinameran.

Supplementary Table 2. Multivariate analysis of antibody response by predefined clinical variables

| Covariate | Anti-RBD-S1 antibody titer at timepoint-3 (log) | | Anti-RBD-S1 antibody titer at timepoint-4 (log) | |
| --- | --- | --- | --- | --- |
|  | Beta (95% CI) | P value | Beta (95% CI) | P value |
| Age (years)  - ≥65 vs. <65 | 0.30 (-0.10 to 0.71) | 0.146 | 0.30 (-0.03 to 0.65) | 0.079 |
| Menopausal status  - postmenopausal vs. prememopausal | -0.47 (-0.94 to 0.01) | 0.053 | -0.41 (-0.81 to -0.01) | 0.044 |
| Smoking habits  - ever vs. never | -0.10 (-0.44 to 0.24) | 0.560 | -0.09 (-0.38 to 0.19) | 0.527 |
| ECOG PS  - 1 vs. 0 | 0.25 (-0.13 to 0.64) | 0.192 | 0.10 (-0.22 to 0.42) | 0.538 |
| BMI  - >25 vs. ≤25 kg/m2 | -0.21 (-0.52 to 0.10) | 0.184 | -0.28 (-0.54 to -0.02) | 0.030 |
| Histology  - ductal vs. lobular | -0.11 (-0.77 to 0.54) | 0.739 | -0.15 (-0.71 to 0.39) | 0.572 |
| Hormone receptor status  - negative vs. positive | -0.29 (-0.93 to 0.33) | 0.357 | -0.32 (-0.85 to 0.21) | 0.239 |
| Ki67 scoring  - ≥20% vs. <20% | 0.17 (-0.34 to 0.69) | 0.518 | 0.27 (-0.15 to 0.71) | 0.211 |
| Treatment setting  - second or later line vs. first line | -0.49 (-0.89 to -0.08) | 0.017 | -0.30 (-0.64 to 0.03) | 0.076 |
| Number of metastatic sites  - ≥2 vs. 1 | 0.20 (-0.20 to 0.61) | 0.323 | 0.04 (-0.29 to 0.38) | 0.778 |
| Visceral involvement  - any vs. not present | 0.15 (-0.22 to 0.54) | 0.657 | -0.21 (-0.53 to 0.11) | 0.200 |
| Previous cytotoxic chemotherapy  - any vs. none | -0.17 (-0.73 to 0.38) | 0.542 | 0.06 (-0.39 to 0.53) | 0.774 |
| Treatment cohort  - CDK4/6 inhibitors vs. anti-HER2 therapies | 0.16 (-0.27 to 0.52) | 0.534 | -0.54 (-0.88 to -0.21) | 0.001 |

RBD-S1, receptor-binding domain (RBD) of the SARS-CoV-2 Spike protein (S1); log, logarithmic value; CI, confidence interval; ECOG PDS, Eastern Cooperative Oncology Group Performance Status; BMI, body mass index; CDK, cyclin-dependent kinase; HER2, epidermal growth factor receptor 2. *P* values derived from parametric 2-sided Wald’s *χ2* test with Bonferroni (α =0.01) correction for multiple comparisons. A two-sided P value of <0.05 was considered statistically significant; previous cytotoxic chemotherapy indicate receipt in the time frame between the first and the third dose tozinameran; timepoint-3 denotes assessment before the third dose of tozinameran; timepoint-4 denotes assessment four weeks after the third dose of tozinameran.

Supplementary Table 3. Multivariate analysis of peripheral lymphocyte counts by predefined clinical variables before the third dose of tozinameran

| Covariate | T helper cell count (log) | | T cytotoxic cell count (log) | | B cell count (log) | | NK cell count (log) | |
| --- | --- | --- | --- | --- | --- | --- | --- | --- |
|  | Beta (95% CI) | P value | Beta (95% CI) | P value | Beta (95% CI) | P value | Beta (95% CI) | P value |
| Age (years)  - ≥65 vs. <65 | 0.02 (-0.08 to 0.14) | 0.626 | 0.01 (-0.14 to 0.16) | 0.927 | 0.05 (-0.10 to 0.21) | 0.488 | 0.13 (-0.01 to 0.28) | 0.067 |
| Menopausal status  - postmenopausal vs. prememopausal | -0.01 (-0.13 to 0.13) | 0.975 | -0.01 (-0.18 to 0.17) | 0.954 | -0.09 (-0.28 to 0.08) | 0.282 | 0.18 (0.01 to 0.35) | 0.033 |
| Smoking habits  - ever vs. never | 0.03 (-0.06 to 0.13) | 0.484 | -0.05 (-0.18 to 0.08) | 0.457 | 0.02 (-0.10 to 0.15) | 0.716 | 0.01 (-0.10 to 0.13) | 0.810 |
| ECOG PS  - 1 vs. 0 | -0.02 (-0.13 to 0.08) | 0.649 | -0.13 (-0.28 to 0.01) | 0.066 | -0.04 (-0.10 to 0.29) | 0.585 | -0.11 (-0.24 to 0.02) | 0.107 |
| BMI  - >25 vs. ≤25 kg/m2 | -0.04 (-0.13 to 0.45) | 0.328 | -0.03 (-0.15 to 0.08) | 0.592 | -0.01 (-0.12 to 0.10) | 0.854 | 0.03 (-0.07 to 0.14) | 0.570 |
| Histology  - ductal vs. lobular | 0.14 (-0.04 to 0.33) | 0.129 | -0.01 (-0.26 to 0.23) | 0.900 | 0.21 (-0.03 to 0.46) | 0.097 | 0.04 (-0.18 to 0.27) | 0.709 |
| Hormone receptor status  - negative vs. positive | 0.02 (-0.12 to 0.16) | 0.790 | 0.13 (-0.10 to 0.37) | 0.273 | 0.12 (-0.12 to 0.36) | 0.324 | 0.05 (-0.16 to 0.28) | 0.606 |
| Ki67 scoring  - ≥20% vs. <20% | 0.02 (-0.12 to 0.16) | 0.790 | 0.02 (-0.16 to 0.22) | 0.777 | -0.06 (-0.26 to 0.13) | 0.500 | -0.01 (-0.18 to 0.18) | 0.981 |
| Treatment setting  - second or later line vs. first line | 0.07 (-0.04 to 0.18) | 0.229 | 0.02 (-0.13 to -0.17) | 0.765 | 0.12 (-0.02 to 0.27) | 0.109 | 0.25 (0.11 to 0.29) | <0.001 |
| Number of metastatic sites  - ≥2 vs. 1 | 0.06 (-0.05 to 0.17) | 0.290 | 0.19 (0.03 to 0.34) | 0.014 | 0.34 (0.15 to 0.45) | <0.001 | 0.09 (-0.05 to 0.23) | 0.210 |
| Visceral involvement  - any vs. not present | 0.14 (0.03 to 0.25) | 0.009 | 0.11 (-0.03 to 0.26) | 0.122 | 0.16 (0.02 to 0.31) | 0.025 | 0.02 (-0.11 to 0.15) | 0.726 |
| Previous cytotoxic chemotherapy  - any vs. none | -0.17 (-0.33 to -0.01) | 0.033 | -0.30 (-0.51 to -0.09) | 0.005 | -0.02 (-0.23 to -0.36) | 0..324 | -0.17 (-0.37 to 0.02) | 0.079 |
| Treatment cohort  - CDK4/6 inhibitors vs. anti-HER2 therapies | -0.23 (-0.34 to -0.12) | <0.001 | -  0.35 (-0.50 to -0.19) | <0.001 | -0.28 (-0.43 to -0.13) | <0.001 | -0.09 (-0.23 to 0.04) | 0.200 |

Log, logarithmic value; CI, confidence interval; ECOG PDS, Eastern Cooperative Oncology Group Performance Status; BMI, body mass index; CDK, cyclin-dependent kinase; HER2, epidermal growth factor receptor 2. *P* values derived from parametric 2-sided Wald’s *χ2* test with Bonferroni (α =0.01) correction for multiple comparisons. A two-sided P value of <0.05 was considered statistically significant. T helper cells, CD3^+^CD4^+^ cells; T cytotoxic cell, CD3^+^CD8^+^; B cells, CD19^+^; NK, Natural killer, CD56^+^CD16^+^; previous cytotoxic chemotherapy indicate receipt in the time frame between the first and the third dose tozinameran.

Supplementary Table 4. Multivariate analysis of peripheral lymphocyte counts by predefined clinical variables after the third dose of tozinameran

| Covariate | T helper cell count (log) | | T cytotoxic cell count (log) | | B cell count (log) | | NK cell count (log) | |
| --- | --- | --- | --- | --- | --- | --- | --- | --- |
|  | Beta (95% CI) | P value | Beta (95% CI) | P value | Beta (95% CI) | P value | Beta (95% CI) | P value |
| Age (years)  - ≥65 vs. <65 | 0.04 (-0.04 to 0.14) | 0.298 | -0.04 (-0.20 to 0.11) | 0.605 | 0.13 (-0.10 to 0.37) | 0.271 | 0.10 (-0.02 to 0.22) | 0.114 |
| Menopausal status  - postmenopausal vs. prememopausal | -0.09 (-0.20 to 0.01) | 0.083 | 0.08 (-0.10 to 0.26) | 0.390 | -0.08 (-0.35 to 0.19) | 0.548 | 0.20 (0.05 to 0.34) | 0.007 |
| Smoking habits  - ever vs. never | -0.03 (-0.10 to 0.04) | 0.454 | -0.06 (-0.20 to 0.06) | 0.322 | -0.06 (-0.26 to 0.13) | 0.395 | -0.13 (-0.23 to -0.02) | 0.014 |
| ECOG PS  - 1 vs. 0 | 0.01 (-0.09 to 0.10) | 0.454 | -0.08 (-0.28 to 0.01) | 0.066 | 0.01 (-0.22 to 0.22) | 0.980 | 0.05 (-0.06 to 0.17) | 0.342 |
| BMI  - >25 vs. ≤25 kg/m2 | -0.02 (-0.07 to 0.09) | 0.848 | -0.03 (-0.23 to 0.06) | 0.322 | 0.05 (-0.12 to 0.23) | 0.581 | 0.07 (-0.02 to 0.17) | 0.120 |
| Histology  - ductal vs. lobular | 0.27 (0.12to 0.42) | <0.001 | 0.15 (-0.10 to 0.41) | 0.240 | 0.27 (-0.12 to 0.63) | 0.185 | 0.08 (-0.11 to 0.28) | 0.417 |
| Hormone receptor status  - negative vs. positive | 0.01 (-0.14 to 0.14) | 0.983 | 0.11 (-0.13 to 0.36) | 0.368 | -0.03 (-0.39 to 0.33) | 0.862 | 0.21 (0.02 to 0.40) | 0.030 |
| Ki67 scoring  - ≥20% vs. <20% | -0.01 (-0.12 to 0.10) | 0.892 | -0.05 (-0.26 to 0.14) | 0.568 | 0.03 (-0.26 to 0.33) | 0.842 | 0.04 (-0.11 to 0.19) | 0.622 |
| Treatment setting  - second or later line vs. first line | 0.02 (-0.06 to 0.11) | 0.634 | -0.01 (-0.15 to 0.15) | 0.985 | -0.06 (-0.29 to 0.17) | 0.603 | 0.10 (0.11 to 0.29) | <0.001 |
| Number of metastatic sites  - ≥2 vs. 1 | 0.03 (-0.09 to 0.12) | 0.504 | 0.06 (-0.09 to 0.22) | 0.436 | 0.12 (-0.11 to 0.35) | 0.304 | 0.09 (-0.01 to 0.23) | 0.088 |
| Visceral involvement  - any vs. not present | 0.04 (-0.04 to 0.12) | 0.332 | 0.13 (-0.01 to 0.28) | 0.086 | 0.16 (-0.06 to 0.37) | 0.167 | -0.03 (-0.15 to 0.08) | 0.570 |
| Previous cytotoxic chemotherapy  - any vs. none | -0.02 (-0.14 to -0.10) | 0.722 | -0.25 (-0.47 to -0.04) | 0.020 | -0.14 (-0.46 to -0.17) | 0.385 | -0.05 (-0.22 to 0.11) | 0.532 |
| Treatment cohort  - CDK4/6 inhibitors vs. anti-HER2 therapies | -0.10 (-0.19 to -0.01) | 0.051 | -  -0.15 (-0.31 to -0.01) | 0.051 | -0.09 (-0.32 to 0.13) | 0.405 | -0.01 (-0.14 to 0.10) | 0.768 |

Log, logarithmic value; CI, confidence interval; ECOG PDS, Eastern Cooperative Oncology Group Performance Status; BMI, body mass index; CDK, cyclin-dependent kinase; HER2, epidermal growth factor receptor 2. *P* values derived from parametric 2-sided Wald’s *χ2* test with Bonferroni (α =0.01) correction for multiple comparisons. A two-sided P value of <0.05 was considered statistically significant. T helper cells, CD3^+^CD4^+^ cells; T cytotoxic cell, CD3^+^CD8^+^; B cells, CD19^+^; NK, Natural killer, CD56^+^CD16^+^; previous cytotoxic chemotherapy indicate receipt in the time frame between the first and the third dose tozinameran.

Supplementary Figure 1. Representative flow cytometry analysis


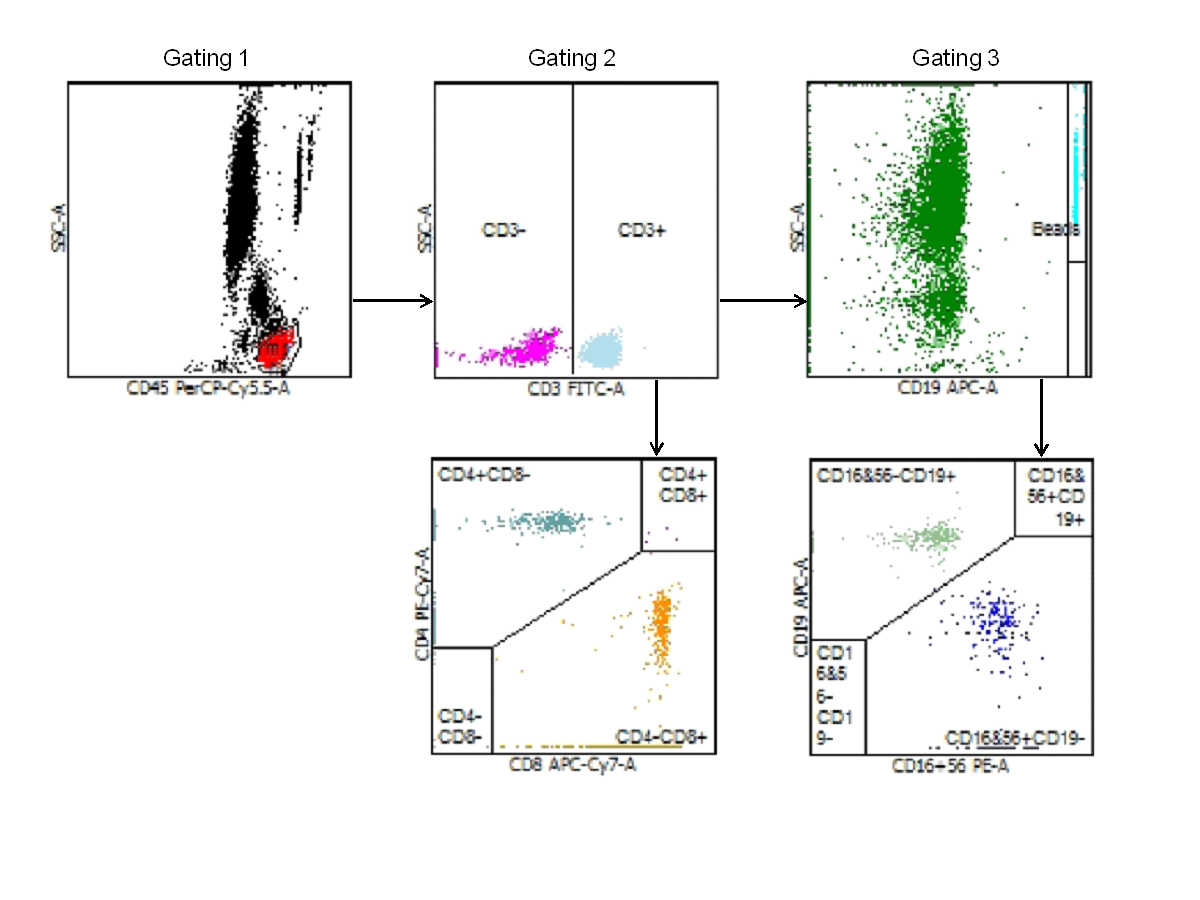


Operating procedures

Three milliliters of whole blood per subject were collected in ethylenediaminetetraacetic acid (EDTA) tubes for flow cytometry analysis. To determine the absolute counts of CD4^+^ and CD8^+^ subpopulations of CD3^+^ T cells, B cells, and NK cells, the BD Multitest 6-color TBNK reagent was utilized. The staining panel consisted of CD3 FITC, CD4 PE-Cy7, CD8 APC-Cy7, CD19 APC, CD45 PerCP-Cy5.5, and CD56 PE + CD16 PE monoclonal antibodies (BD Biosciences, San Jose, CA). BD Trucount tubes, also from BD Biosciences, were loaded with 20 µL of the BD Multitest 6-color TBNK reagent and 50 µL aliquots of EDTA-anticoagulated whole blood. After incubating the mixture in the dark at room temperature for 20 minutes, it was lysed with 2 mL of FACS Lysis Solution from BD Biosciences. Following an additional 15 minutes of incubation, the erythrocyte-lysed, unwashed, and stained samples were analyzed. The BD FACSCanto II system and BD FACSCanto clinical software, both from BD Biosciences, were used for data acquisition. The instrument was calibrated with BD FACS 7-color setup beads before each running process, as per the manufacturer's instructions [1]. The results for each lymphocyte subset were reported as absolute cell counts/µL.

Gating strategy

The first gating involves analyzing forward scatter (CD45^+^) and side scatter (SSC-A) to identify the total number of lymphocytes. The second gating focuses on forward scatter (CD3^+^) and side scatter (SSC-A) to determine the total number of T lymphocytes, and then further examines CD8^+^ and CD4^+^ to identify the absolute values of T helper cells (CD3^+^CD4^+^) and T cytotoxic cells (CD3^+^CD8^+^). Lastly, the third gating analyzes forward scatter (CD19^+^) and side scatter (SSC-A) to detect the total number of B lymphocytes (CD19^+^), and then examines CD56^+^CD16^+^ and CD19^+^ to determine the absolute values of NK cells (CD56^+^CD16^+^).

References

1. BD FACSCanto™ Software. https://www.bdbiosciences.com/en-eu/products/software/instrument-software/bd-facscanto-clinical-software/; [Accessed October 12, 2023].
